# Autoimmune Hepatitis Induced by Immune Checkpoint Inhibitors in Adults: A Systematic Review

**DOI:** 10.3390/diagnostics16121821

**Published:** 2026-06-12

**Authors:** Sarita Chonat, Jonathan Soldera

**Affiliations:** 1 Acute Medicine, University of South Wales in Association with Learna Ltd., Cardiff CF37 1DL, UK; drsaritachonat@gmail.com; 2 Gastroenterology and Acute Medicine, University of South Wales in Association with Learna Ltd., Cardiff CF37 1DL, UK; 3 Logan Hospital, Brisbane, QLD 4131, Australia

**Keywords:** immune checkpoint inhibitors, hepatitis, autoimmune, drug-induced liver injury, immunotherapy, mycophenolic acid

## Abstract

**Background/Objectives:** Immune checkpoint inhibitors (ICIs) have changed the treatment landscape for several advanced malignancies, but their use is accompanied by immune-related adverse events, including liver injury. Some cases resemble autoimmune hepatitis (AIH), although many are more accurately described as AIH-like immune-mediated hepatitis rather than classical AIH. This distinction matters, as diagnosis is often based on exclusion and management must balance hepatic recovery against interruption of potentially life-prolonging cancer therapy. This systematic review summarised the clinical phenotype, diagnostic assessment, treatment strategies, treatment response, ICI discontinuation, and rechallenge outcomes in patients with ICI-associated AIH-like liver injury. **Methods:** A systematic PubMed search was performed for English-language human studies reporting autoimmune hepatitis, AIH-like liver injury, or immune-mediated hepatitis following exposure to ICIs. Eligible studies included case reports, case series, retrospective cohorts, prospective cohorts, and pharmacovigilance-type studies with extractable clinical, treatment, or outcome data. Reviews, guidelines, non-original articles, animal studies, non-English publications, and reports without usable liver injury data were excluded. The review followed PRISMA principles. Risk of bias was assessed using Joanna Briggs Institute tools and summarised with ROBVIS. Given the heterogeneity of study design, diagnostic criteria, treatment definitions, and outcome reporting, formal meta-analysis was not appropriate; results were therefore synthesised descriptively. **Results:** Twenty-two studies were included, comprising 195 patients with ICI-associated AIH-like or immune-mediated hepatitis. Of these, 140 patients received active treatment, and 133/140 achieved clinical or biochemical recovery with varying therapies. Corticosteroids were the most frequently used first-line therapy, with recovery reported in 102/105 patients treated with corticosteroids alone. Mycophenolate mofetil was the main second-line agent for steroid-refractory disease, with response reported in 9/10 treated patients. Other therapies, including tacrolimus, azathioprine, ursodeoxycholic acid, bezafibrate, tocilizumab, basiliximab, infliximab, budesonide, and double plasma molecular adsorption system with or without plasma exchange, were described only in small numbers or isolated cases. Spontaneous recovery without pharmacological treatment was reported in 19 patients. ICI interruption or discontinuation occurred in 141 patients, and rechallenge was reported in 55 patients after recovery, with no recurrent hepatic toxicity documented in the extracted dataset. **Conclusions:** ICI-associated AIH-like liver injury is an important immune-related toxicity, but the available literature remains fragmented and methodologically heterogeneous. Most reported patients recovered, particularly with corticosteroids, and MMF appears to be the most consistently used escalation therapy in steroid-refractory cases. However, the strength of evidence is limited by uncontrolled designs, variable terminology, inconsistent diagnostic work-up, and non-standardised outcome definitions. Future studies should separate classical AIH from AIH-like immune-mediated hepatitis, use uniform criteria for severity and response, and report treatment denominators clearly, especially for rechallenge and steroid-refractory disease.

## 1. Introduction

Immune checkpoint inhibitors (ICIs) have changed the treatment of advanced malignancies and are now central to the management of several tumour types. By restoring antitumour immune activity, these agents have improved outcomes in cancers such as melanoma, non-small cell lung cancer, renal cell carcinoma, and hepatocellular carcinoma [1,2,3,4,5]. Their effect is mediated through blockade of inhibitory immune pathways, particularly cytotoxic T-lymphocyte-associated protein 4 (CTLA-4), programmedcell death protein 1 (PD-1), and programmed death-ligand 1 (PD-L1), which normally restrain T-cell activation and maintain immune tolerance [6,7,8]. Ipilimumab, a CTLA-4 inhibitor, was the first ICI approved by the U.S. Food and Drug Administration for metastatic melanoma in 2011 [9]. Since then, PD-1 and PD-L1 inhibitors, including nivolumab, pembrolizumab, atezolizumab, avelumab, and durvalumab, have been incorporated into treatment pathways across multiple cancers [10,11,12,13].

This therapeutic gain has come with a predictable cost. By lowering immune tolerance, ICIs may cause immune-related adverse events (irAEs), affecting the skin, gastrointestinal tract, endocrine organs, lungs, liver, and other systems [14,15,16,17]. Most irAEs are mild or manageable, but some are clinically serious and may require treatment interruption, corticosteroids, second-line immunosuppression, or permanent discontinuation of cancer therapy. Fatal events are uncommon, but the burden of toxicity is real, particularly when hepatic injury creates uncertainty over whether immunotherapy can be safely continued or restarted [18,19,20,21].

Hepatic irAEs are usually described as immune-mediated hepatitis (IMH), ICI-induced liver injury, or autoimmune hepatitis (AIH)-like liver injury [22,23,24,25]. The liver is particularly vulnerable to disturbances in immune tolerance because of its constant exposure to gut-derived antigens through the portal circulation. Hepatic immune homeostasis depends partly on checkpoint pathways, including PD-1/PD-L1 and CTLA-4 signalling, expressed through Kupffer cells, stellate cells, dendritic cells, and regulatory T-cell networks [26,27,28,29]. When these inhibitory pathways are blocked, antitumour immunity may improve, but hepatic self-tolerance may also be disrupted, resulting in inflammatory hepatocellular injury [30,31,32].

The frequency and severity of hepatic toxicity vary according to ICI class and treatment regimen. Anti-PD-1 or anti-PD-L1 monotherapy is associated with hepatotoxicity in approximately 3–16% of patients, while anti-CTLA-4 therapy appears to carry a higher risk of grade ≥ 3 hepatitis, reported in up to 12% of cases [33,34,35,36]. Combination ICI therapy increases this risk further, with liver enzyme elevation reported in up to 37% of patients in some series [37,38,39]. Risk is also influenced by tumour type, combination regimens, baseline liver disease, concurrent autoimmune disease, and possibly the occurrence of irAEs in other organs [40,41,42,43].

Most patients with ICI-associated hepatitis are asymptomatic, and the diagnosis is often triggered by routine blood tests showing elevated aminotransferases before a scheduled treatment cycle [44,45]. A smaller proportion develop fatigue, abdominal pain, fever, rash, jaundice, or other non-specific symptoms. Acute liver failure can occur, but it is rare [46]. The timing is variable, although many cases occur within 6–14 weeks of starting immunotherapy. Delayed immune-related events may also occur months after ICI discontinuation, which can make attribution more difficult [47]. Biochemically, the pattern may be hepatocellular, cholestatic, or mixed, and some patients evolve from a mixed pattern early in the course to a more hepatocellular picture at peak injury [48].

Diagnosis remains difficult because ICI-related hepatitis overlaps with several common and important alternatives, including classical autoimmune hepatitis, viral hepatitis, drug-induced liver injury, alcohol-related liver disease, biliary obstruction, hepatic metastases, sepsis, and vascular liver disease [49,50,51]. There is no single diagnostic serological marker. For this reason, ICI-hepatitis is usually a diagnosis of exclusion, supported by temporal association with ICI exposure and exclusion of competing causes. Work-up should include viral serology, autoimmune markers, medication review, assessment for other systemic illness, and imaging of the liver, biliary tree, and hepatic vasculature where appropriate [52,53,54]. Severity is commonly graded using the Common Terminology Criteria for Adverse Events (CTCAE), but this system is imperfect in liver disease because it is driven largely by aminotransferases and bilirubin and does not fully incorporate markers of hepatic function such as INR or prothrombin time [55].

Liver biopsy is not required in every case, but it is useful when the diagnosis is uncertain, when liver tests fail to improve as expected, when competing diagnoses remain plausible, or when underlying liver disease may alter management. Histology is not pathognomonic. Anti-PD-1 and anti-PD-L1 injury more often shows lobular hepatitis with lymphocytic and histiocytic inflammation and sometimes bile duct injury, while anti-CTLA-4 therapy has been associated more often with granulomatous hepatitis [56,57,58]. Compared with classical AIH or conventional drug-induced liver injury, ICI-related hepatitis usually has fewer plasma cells and eosinophils, and immunohistochemistry often shows a predominance of CD3+ and CD8+ T cells [59,60]. These features support the concept that many cases are AIH-like rather than true classical autoimmune hepatitis.

The terminology used in the literature remains inconsistent. Reports variously describe immune-related hepatitis, immune-mediated hepatotoxicity, hepatic irAEs, ICI-induced liver injury, autoimmune-like hepatitis, dysimmune hepatitis, transaminitis, and autoimmune hepatitis [61]. This is not merely semantic. Labelling all cases as autoimmune hepatitis risks implying a disease phenotype identical to classical AIH, when many patients lack typical autoantibodies, elevated IgG, plasma-cell-rich interface hepatitis, or chronic relapsing behaviour. A more careful term, such as ICI-associated AIH-like immune-mediated hepatitis, may better reflect the current evidence and the diagnostic uncertainty that clinicians face.

Management should be guided by severity, clinical trajectory, symptoms, liver function, and the need to preserve oncological benefit. Corticosteroids remain the usual first-line treatment for clinically significant hepatitis, although practice varies for grade 2 disease and for asymptomatic patients with marked aminotransferase elevation but preserved bilirubin and INR [44,50]. In steroid-refractory cases, mycophenolate mofetil is the most commonly recommended second-line agent. Other approaches, including tacrolimus, azathioprine, tocilizumab, basiliximab, budesonide, ursodeoxycholic acid, bezafibrate, infliximab, and extracorporeal liver-support strategies, have been reported, but the evidence is limited and often restricted to isolated cases or small series. Predicting steroid non-response remains difficult, although hyperbilirubinemia and underlying liver disease may indicate a more complicated course [4]. This uncertainty has direct clinical consequences. ICI-related liver injury may interrupt or permanently stop otherwise effective cancer therapy, while excessive or prolonged immunosuppression may increase infection risk, metabolic complications, and possibly weaken anticancer immune activity. Rechallenge after recovery remains unresolved: some patients restart the same or a different ICI without recurrent hepatitis, whereas others relapse. Current evidence comes mainly from retrospective cohorts, pharmacovigilance analyses, case series, and case reports, with no large randomised trials defining the optimal diagnostic threshold, steroid regimen, second-line therapy, or rechallenge strategy [40,44]. Novel approaches, including double plasma molecular adsorption system (DPMAS) for severe or refractory presentations, have been described, but remain exploratory and require cautious interpretation [27,28].This systematic review aims to synthesise the available evidence on ICI-associated AIH-like immune-mediated hepatitis in adults, focusing on clinical presentation, severity, diagnostic assessment, histological phenotype, treatment strategies, response to therapy, ICI discontinuation, rechallenge, and reported outcomes. Given the heterogeneity of definitions, study designs, denominators, and outcome measures, the review is presented as a descriptive systematic review rather than a formal meta-analysis, with the goal of clarifying what can reasonably be concluded from the current literature and where uncertainty remains.

## 2. Materials and Methods

### 2.1. Protocol and Registration

This systematic review was conducted in accordance with the Preferred Reporting Items for Systematic Reviews and Meta-Analyses (PRISMA) 2020 guidance. The protocol was defined before data extraction and specified the review question, eligibility criteria, search strategy, outcomes of interest, and planned method of synthesis. The protocol was registered in the International Prospective Register of Systematic Reviews (PROSPERO; registration number: CRD420251075057).

### 2.2. Search Strategy

A systematic search of PubMed/MEDLINE was performed to identify studies reporting autoimmune hepatitis (AIH), AIH-like liver injury, or immune-mediated hepatitis (IMH) occurring after exposure to immune checkpoint inhibitors (ICIs). The search was last updated on 18 April 2025. The strategy combined Medical Subject Headings (MeSH) and free-text terms related to autoimmune liver injury, drug-induced liver injury, and immune checkpoint blockade. The full search strategy was:

*(“Hepatitis, Autoimmune”[MeSH] OR “Liver Toxicity”[TIAB] OR “DILI”[TIAB] OR “Autoimmune Hepatitis”[TIAB] OR “Liver Injury”[TIAB]) AND (“Immune Checkpoint Inhibitors”[MeSH] OR “Immune Checkpoint Blockade”[TIAB] OR “PD-1 Inhibitors”[TIAB] OR “CTLA-4 Inhibitors”[TIAB] OR “PD-L1 Inhibitors”[TIAB] OR “Immune Checkpoint”[TIAB])*.

No date restriction was applied. Only English-language publications involving human participants were considered. Records were manually screened first by title and abstract, followed by full-text assessment of potentially eligible reports. Duplicate or overlapping reports were manually removed during screening. When a study appeared potentially relevant but the available information was insufficient, the full text was sought before a final decision was made.

### 2.3. Eligibility Criteria

Eligibility was structured using the PICOS framework. Given the inconsistent terminology in this field, we did not restrict inclusion to cases fulfilling formal criteria for classical autoimmune hepatitis. Studies were eligible when the original authors described ICI-associated autoimmune hepatitis, AIH-like liver injury, immune-mediated hepatitis, immune-related hepatotoxicity, or ICI-induced liver injury with sufficient clinical detail for extraction. The terminology used by each study was retained during data extraction, and cases were interpreted within the broader framework of ICI-associated AIH-like/immune-mediated hepatitis unless classical AIH features were clearly documented.

Population (P): Adults diagnosed with autoimmune hepatitis, AIH-like liver injury, immune-mediated hepatitis, or immune-related liver injury after exposure to ICIs.

Intervention/Exposure (I): Treatment with ICIs targeting PD-1, PD-L1, CTLA-4, or combination checkpoint blockade, including agents such as nivolumab, pembrolizumab, atezolizumab, durvalumab, ipilimumab, and related drugs.

Comparison (C): No formal comparison group was required. Studies were eligible if they reported extractable data on ICI-associated liver injury, even when no control arm was present. Where studies included comparisons with non-ICI liver injury, baseline liver tests, or alternative clinical groups, these data were recorded descriptively but were not treated as formal comparators.

Outcomes (O): Eligible outcomes included clinical presentation, biochemical pattern, severity grading, diagnostic work-up, autoantibodies, IgG, liver histology, treatment used, treatment response, biochemical or clinical resolution, ICI interruption or discontinuation, rechallenge, recurrence, steroid-refractory disease, liver-related mortality, and other severe outcomes.

Study Design (S): Primary clinical studies were eligible, including case reports, case series, retrospective cohorts, prospective cohorts, observational studies, and pharmacovigilance-type studies. Reviews, systematic reviews, meta-analyses, editorials, guidelines, non-human studies, and reports without extractable liver injury or outcome data were excluded.

### 2.4. Inclusion and Exclusion Criteria

Studies were included if they reported original clinical data on AIH, AIH-like liver injury, immune-mediated hepatitis, or immune-related hepatic toxicity associated with ICI exposure. The diagnosis could be based on clinical assessment, biochemical abnormalities, exclusion of alternative causes, histology, or the diagnostic criteria used by the original study. Studies were also required to provide sufficient information to extract at least one relevant clinical, diagnostic, treatment, or outcome variable.

Because the terminology in the literature is heterogeneous, we did not require included cases to fulfil formal diagnostic criteria for classical autoimmune hepatitis. Studies were eligible when the original authors reported autoimmune hepatitis, AIH-like liver injury, immune-mediated hepatitis, immune-related hepatotoxicity, or ICI-induced liver injury with sufficient clinical detail to support attribution to ICI exposure. During extraction, the terminology used by each study was retained, and cases were interpreted under the broader framework of ICI-associated AIH-like/immune-mediated hepatitis unless classical AIH features were explicitly documented. This approach was chosen to avoid excluding clinically relevant reports while still acknowledging that the included studies may not represent a single uniform disease entity.

Studies were excluded if they were reviews, meta-analyses, editorials, expert opinions, clinical guidelines, non-English publications, animal studies, duplicate reports, or reports without sufficient patient-level or study-level data. Studies focused only on non-hepatic irAEs were excluded. Studies describing liver enzyme abnormalities without enough information to attribute them to ICI-related hepatitis or AIH-like injury were also excluded.

### 2.5. Data Collection and Extraction

Data were extracted independently by two reviewers using a predefined extraction form. Disagreements were resolved by discussion and consensus. Extracted variables included first author, year of publication, country or region, study design, number of patients, cancer type, ICI agent and class, use of monotherapy or combination therapy, time to liver injury where available, biochemical pattern, severity grade, diagnostic work-up, autoimmune markers, IgG, liver biopsy findings, treatment used, treatment response, biochemical or clinical recovery, ICI interruption or discontinuation, rechallenge, recurrence after rechallenge, and mortality or other severe outcomes.

Treatment data were extracted by specific therapy whenever possible. These included ICI interruption or withdrawal, corticosteroids, mycophenolate mofetil, azathioprine, tacrolimus, ursodeoxycholic acid, bezafibrate, infliximab, tocilizumab, basiliximab, budesonide, plasma exchange, double plasma molecular adsorption system, and other second-line or rescue therapies. Response was extracted preferentially as the number responding over the number treated. Percentages alone were used only when absolute numbers could be derived or when no other data were available.

### 2.6. Risk of Bias Assessment

Risk of bias and methodological quality were assessed using the Joanna Briggs Institute critical appraisal tools, selected according to study design. Case reports, case series, prevalence-type studies, and observational studies were assessed with the most appropriate JBI checklist. The results were summarised descriptively and visualised using ROBVIS. Risk of bias assessment was used to contextualise the strength of the evidence, rather than to exclude studies automatically, given the rarity of the condition and the predominance of uncontrolled designs.

### 2.7. Data Synthesis

A formal meta-analysis was not performed. This decision was made before final synthesis because the included studies were heterogeneous in study design, diagnostic terminology, ICI exposure, cancer type, severity grading, diagnostic work-up, treatment regimen, follow-up duration, and outcome definitions. Most reports did not include true comparator groups, and several outcomes lacked consistent denominators.

The findings were therefore synthesised descriptively. Frequencies and proportions were calculated where denominators were clear. Results were organised around study characteristics, implicated ICI agents, cancer types, clinical and biochemical phenotype, diagnostic assessment, histology, management, treatment response, ICI discontinuation, rechallenge, recurrence, and severe outcomes. Narrative synthesis was used when numerical pooling would have been misleading.

### 2.8. Statistical Analysis

Quantitative analysis was limited to descriptive statistics. The following variables were calculated where data allowed: total number of ICI-associated AIH-like or immune-mediated hepatitis cases, number of patients receiving each treatment, response by treatment group, overall recovery, spontaneous resolution without pharmacological treatment, ICI interruption or discontinuation, ICI rechallenge, recurrence after rechallenge, and liver-related mortality or severe hepatic outcomes.

Treatment response was reported as n/N whenever possible. Percentages were used only to support absolute numbers and not as a substitute for denominators. No inferential testing, comparative modelling, or pooled effect estimates were performed because the available data did not meet the assumptions required for formal meta-analysis.

## 3. Results

### 3.1. Literature Search and Study Selection

The PubMed search retrieved 277 records. After duplicate removal and initial screening, 52 records were considered potentially relevant and assessed in more detail. Studies were then excluded if they were reviews, guidelines, non-English publications, duplicate or overlapping reports, unrelated to ICI-associated liver injury, or did not provide extractable clinical or outcome data. One potentially eligible study was not publicly available in full text, and the corresponding authors were contacted. In total, 22 studies met the inclusion criteria and were included in the final synthesis.

The final dataset included 195 patients with ICI-associated autoimmune hepatitis-like liver injury or immune-mediated hepatitis. The evidence base was mainly observational and descriptive: 12 studies were observational cohorts or cohort-type studies, and 10 were case reports or case series. No randomised controlled trial was identified. This limited the analysis to descriptive synthesis rather than formal meta-analysis.

**Figure 1:**
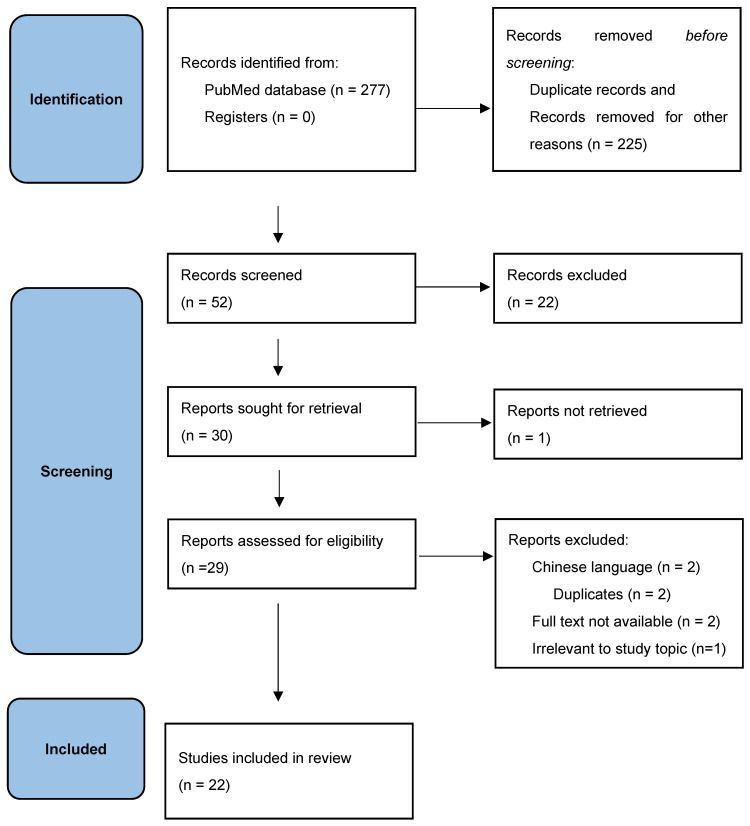
PRISMA flow diagram of study selection [62].

Among the 195 included patients, 140 received active treatment for ICI-associated hepatitis. Clinical or biochemical recovery was reported in 133/140 treated patients with varying regimens. Spontaneous recovery without pharmacological treatment was described in 19 patients. ICI interruption or discontinuation was reported in 141 patients. Rechallenge was attempted in 55 patients after improvement or resolution of liver injury, and no recurrent hepatic toxicity was documented in those rechallenged in the extracted dataset. These figures should be interpreted cautiously, as definitions of hepatitis, response, recovery, and rechallenge varied across studies. The study selection process is shown in Figure 1.

### 3.2. Risk of Bias Assessment

Risk of bias was assessed using the appropriate Joanna Briggs Institute tools according to study design and summarised using ROBVIS. Overall, the case reports were generally adequate for diagnostic description, treatment chronology, and short-term outcome reporting [2,5,6,7,11,12,15,16,18,21]. Their main limitations were predictable: small numbers, incomplete follow-up in some cases, and limited ability to separate treatment effect from the natural course of the liver injury. This is available in the Supplementary Materials (Tables S1–S7).

The observational studies provided broader information on frequency, treatment patterns, severity, and rechallenge, but they were also limited by retrospective design, variable diagnostic definitions, and inconsistent outcome reporting [1,3,4,8,9,10,13,14,17,19,20,22]. Some studies included all immune-related hepatotoxicity rather than strictly classical autoimmune hepatitis, while others used autoimmune hepatitis terminology for cases that were more accurately AIH-like or immune-mediated. For this reason, the risk of bias assessment supported inclusion for descriptive purposes but did not justify pooled comparative estimates.

### 3.3. Characteristics of Included Studies

The 22 included studies covered a broad range of malignancies, ICI classes, clinical phenotypes, and treatment approaches [1,2,3,4,5,6,7,8,9,10,11,12,13,14,15,16,17,18,19,20,21,22]. Patients had been exposed to PD-1 inhibitors, PD-L1 inhibitors, CTLA-4 inhibitors, or combination checkpoint blockade. The most commonly reported agents included nivolumab, pembrolizumab, atezolizumab, durvalumab, and ipilimumab. The underlying cancers were heterogeneous and included melanoma, non-small cell lung cancer, renal cell carcinoma, hepatobiliary malignancies, oesophageal cancer, and other advanced tumours.

The included studies differed substantially in the way liver injury was defined. Some reports described autoimmune hepatitis, some immune-mediated hepatitis, and others immune-related hepatotoxicity or ICI-induced liver injury. The extent of diagnostic work-up also varied. Some studies included liver biopsy, autoantibodies, and exclusion of competing causes, whereas others relied mainly on liver biochemistry, clinical course, and temporal association with ICI exposure. This heterogeneity was the main reason the results were analysed descriptively. They are described in Table 1; more complete data is available in the Supplementary Materials.

### 3.4. Case Reports and Case Series

The case reports and small case series provided the most detailed clinical descriptions, particularly for severe, delayed, or steroid-refractory presentations. They also captured treatment approaches that are unlikely to appear in larger cohorts because they are used only in selected refractory cases.

Zarrabi et al. reported severe hepatitis after nivolumab for refractory osteosarcoma [18]. The patient did not respond adequately to conventional treatment with corticosteroids and other immunosuppressive strategies, but improved after basiliximab [18]. Parakh et al. described delayed hepatitis occurring eight months after nivolumab discontinuation, which responded to corticosteroid therapy, highlighting that ICI-associated liver injury may present well after the last dose [12].

Ziogas et al. reported severe steroid-resistant hepatitis after ipilimumab for metastatic melanoma, which required triple immunosuppression with corticosteroids, mycophenolate mofetil, and tacrolimus, with subsequent biochemical resolution [21]. Onishi et al. described a steroid-refractory or incompletely steroid-responsive case after nivolumab, in which ursodeoxycholic acid and bezafibrate were added with improvement in liver tests [11].

Feng et al. described a patient treated with combined nivolumab and ipilimumab for advanced oesophageal squamous cell carcinoma who developed hepatitis requiring corticosteroids, whose clinical course was complicated by opportunistic infections, including herpes zoster and tuberculosis, requiring withdrawal of corticosteroid therapy and leaving persistent grade 2 liver dysfunction [5].

Honma et al. reported hepatitis after atezolizumab-based therapy with bevacizumab, carboplatin, and paclitaxel, whose treatment with oral prednisolone and ursodeoxycholic acid was associated with improvement in liver biochemistry [6]. Tan et al. described acute liver failure after camrelizumab-based therapy in a patient with oesophageal cancer, managed with dual plasma molecular adsorption system and plasma exchange [16]. Shah et al. reported complete biochemical recovery with high-dose corticosteroids and mycophenolate mofetil in a patient who developed hepatitis after pembrolizumab [15].

Across these individual reports, the clinical course was usually favourable when the injury was recognised and treated promptly. However, the value of these cases lies less in estimating response rates and more in showing the range of presentations and rescue strategies used when first-line corticosteroids were inadequate or unsafe [2,5,6,7,11,12,15,16,18,21].

### 3.5. Observational Studies

The observational studies contributed most of the patient numbers and were the main source for treatment frequency, recovery, ICI discontinuation, and rechallenge outcomes. De Martin et al. reported a retrospective French cohort of patients who developed liver injury after ICI therapy across several malignancies, including melanoma, non-small cell lung cancer, and renal cell carcinoma [4]. Patients had received anti-PD-1, anti-PD-L1, anti-CTLA-4 therapy, or combination therapy [4]. Most patients improved with corticosteroids, while two required additional immunosuppression before resolution [4].

Kocheise et al. examined ICI use in patients with autoimmune liver disease in a multicentre cohort, including patients with primary biliary cholangitis, primary sclerosing cholangitis, autoimmune hepatitis, and overlap phenotypes [8]. Although this population differs from patients who develop de novo ICI-hepatitis, the study is relevant because it addresses the safety of ICI exposure in a group usually excluded from clinical trials [8]. In the extracted ICI-hepatitis cases, management was individualised and recovery was reported [8].

Zhang et al. provided the largest source of severe hepatitis data [20]. In this multicentre Chinese cohort, 5326 patients received ICIs, 186 developed immune-related hepatotoxicity, and 51 developed grade ≥ 3 hepatitis [20]. Corticosteroids were used as first-line therapy [20]. Escalation was required in selected cases, including mycophenolate mofetil, tacrolimus, and tocilizumab [20]. This study was particularly useful for severe immune-related hepatotoxicity, although not all cases necessarily fulfilled a strict classical AIH phenotype [20].

Jilkova et al. contributed histological and comparative data, rather than treatmentresponse data [22]. The study compared ICI-associated hepatitis with autoimmune liver disease and supported the concept that ICI-hepatitis may overlap with, but is not identical to, classical autoimmune hepatitis [22].

Riveiro-Barciela et al. reported a prospective multicentre cohort of patients who had experienced severe immune-related hepatitis and discontinued immunotherapy [13]. Nineteen patients received corticosteroids, including oral prednisone or methylprednisolone, and two required mycophenolate mofetil as second-line therapy [13]. This cohort was also central to the rechallenge analysis, as several patients restarted immunotherapy after liver injury resolved [13].

Luo et al. focused on patients with advanced lung cancer who developed steroid-refractory immune-related adverse events after ICI therapy [9]. Six patients with hepatitis received mycophenolate mofetil in addition to corticosteroids, and five improved within three months [9]. This study was clinically important because it addressed second-line immunosuppression in steroid-refractory disease [9].

Sanz-Segura et al. reported immune-mediated hepatitis in patients with advanced cancers treated with nivolumab, pembrolizumab, atezolizumab, durvalumab, ipilimumab, or combination therapy [14]. Among four patients with immune-mediated hepatitis, two required corticosteroids and one achieved complete recovery [14]. Yamamoto et al. reported patients with elevated transaminases during ICI therapy, including exposure to ipilimumab, nivolumab, pembrolizumab, and atezolizumab [17]. Thirteen patients received corticosteroids and all responded [17].

Coukos et al. compared ICI-induced liver injury with autoimmune liver diseases in a multicentre cohort [3]. Fourteen patients with ICI-associated hepatitis received corticosteroids alone or corticosteroids with additional immunosuppression, and all achieved recovery [3]. Zen et al. provided detailed histological assessment of liver dysfunction after nivolumab or ipilimumab; five of six patients improved after corticosteroid treatment [19]. Purde et al. reported a prospective multicentre cohort in which six steroid-treated patients achieved biochemical recovery [10]. Araujo et al. contributed one case of immune-mediated hepatitis treated with infliximab in the context of steroid-refractory immune-related adverse events, with reported improvement [1].

Taken together, the observational data support three main results: most reported patients improved, corticosteroids were the dominant first-line therapy, and second-line immunosuppression was reserved for steroid-refractory or severe cases [1,3,4,8,9,10,13,14,17,19,20,22]. However, treatment thresholds and response definitions were not uniform across studies.

### 3.6. Treatment Outcomes

Treatment data were available for 140 patients. Corticosteroids were the most commonly used treatment and were reported as monotherapy in 105 patients [3,4,6,10,12,13,14,17,19,20]. Recovery occurred in 102/105 patients treated with corticosteroids alone. This was the largest treatment group and therefore provides the most stable estimate in the dataset.

Mycophenolate mofetil was the most frequently used second-line agent [4,9,13,15,20,21]. Among patients treated with corticosteroids and mycophenolate mofetil, recovery was reported in 9/10 [4,9,13,15,20]. A further 3 patients received corticosteroids, mycophenolate mofetil, and tacrolimus, with recovery in all 3 [20,21]. Other agents were reported in much smaller numbers. These included azathioprine, tacrolimus, tocilizumab, infliximab, basiliximab, ursodeoxycholic acid, bezafibrate, budesonide, and extracorporeal liver-support strategies such as double plasma molecular adsorption system with or without plasma exchange [1,2,7,8,11,16,18,20,21].

The apparent high response rates for several of these therapies should not be overinterpreted. Most were reported in one or two patients, usually in selected severe or refractory cases. Tocilizumab was the only therapy in this extracted dataset with a visibly lower response proportion, with recovery in 4/7 treated patients [20]. Basiliximab and DPMAS-based therapy showed favourable outcomes, but only in isolated cases [7,16,18].

Because definitions of response were not uniform across the included studies, these proportions should be read as descriptive treatment outcomes rather than pooled efficacy estimates. Recovery variably referred to biochemical improvement, biochemical normalisation, clinical resolution, or improvement sufficient to permit corticosteroid tapering or ICI rechallenge, depending on the original study. The denominator for each treatment group was also small for several interventions, particularly rescue therapies and biologics. For this reason, the values in Table 2 are intended to summarise the reported clinical experience, not to compare treatment efficacy across modalities.

### 3.7. ICI Discontinuation, Spontaneous Resolution, and Rechallenge

ICI interruption or discontinuation was common and was reported in 141/195 patients [1,2,3,4,5,6,7,9,10,12,13,15,16,17,18,19,20,21]. In many studies, treatment was stopped at the time of grade ≥ 3 hepatitis or when liver injury was considered clinically significant. However, the indication for discontinuation was not always clearly separated from the decision to start corticosteroids. Therefore, discontinuation rates should be read as a marker of clinical caution and severity, not as proof that permanent ICI withdrawal is always required.

Spontaneous resolution without pharmacological treatment was reported in 19 patients [3,4,10,13,19]. This finding is important because it suggests that not all biochemical hepatitis after ICI exposure follows the same trajectory. Some patients, particularly those without jaundice, synthetic dysfunction, or progressive worsening, may improve with ICI interruption and close monitoring alone. The studies did not provide enough uniform data to define which patients can be safely managed without immunosuppression.

ICI rechallenge was reported in 55 patients [4,9,10,13,20]. In the extracted dataset, rechallenge with the same or a different ICI was not associated with documented recurrent hepatic toxicity. This finding is encouraging, but it should be interpreted carefully. Patients selected for rechallenge were likely clinically stable, had recovered from the initial event, and were considered suitable by treating teams. The available data do not allow estimation of rechallenge safety in unselected patients or in those with severe steroid-refractory hepatitis.

Overall, the results show a condition with generally favourable hepatic outcomes when recognised and managed, but with substantial uncertainty around nomenclature, diagnostic thresholds, treatment escalation, and rechallenge decisions. The evidence supports corticosteroids as first-line treatment for clinically significant cases and mycophenolate mofetil as the most commonly used second-line therapy, while the role of other agents remains based on limited descriptive evidence.

## 4. Discussion

In this systematic review, 22 studies comprising 195 patients with ICI-associated autoimmune hepatitis-like liver injury or immune-mediated hepatitis were included. Among 140 patients who received active treatment, 133 achieved clinical or biochemical recovery with varying treatment regimens. Corticosteroids were the dominant first-line therapy and were associated with recovery in 102/105 patients treated with steroids alone. Mycophenolate mofetil was the most frequently used second-line agent, with response in 9/10 patients treated with corticosteroids and MMF. Other therapies, including tacrolimus, azathioprine, tocilizumab, infliximab, basiliximab, budesonide, ursodeoxycholic acid, bezafibrate, plasma exchange, and DPMAS, were reported in much smaller numbers and mostly in refractory or severe cases. Spontaneous recovery occurred in 19 patients, while ICI discontinuation was reported in 141 patients and rechallenge in 55. These findings show that hepatic outcomes are usually favourable when the condition is recognised and managed, but they also show how limited the evidence remains. The available literature is not a clean therapeutic dataset. It is a mixed body of case reports, retrospective cohorts, prospective observational studies, and pharmacovigilance-type reports, with variable definitions and uneven reporting.

The first point emerging from these data is that ICI-associated hepatitis should not be treated as identical to classical autoimmune hepatitis. The terminology in the literature remains imprecise, with the same condition described as autoimmune hepatitis, autoimmune-like hepatitis, immune-mediated hepatitis, immune-related hepatotoxicity, hepatic irAE, or ICI-induced liver injury. This is more than a writing issue. Classical AIH has established diagnostic expectations, including autoantibodies, IgG elevation, compatible histology, exclusion of competing causes, and a chronic relapsing tendency. Many ICI-associated cases do not fulfil this phenotype. Some have negative autoantibodies, absent or modest IgG elevation, lobular rather than interface-predominant hepatitis, and a monophasic course after ICI withdrawal or immunosuppression. Histological studies comparing ICI-related hepatitis with classical autoimmune liver disease support this distinction, showing overlapping but not identical patterns, with lobular injury, CD8-predominant inflammation, granulomatous features in some anti-CTLA-4 cases, and fewer plasma cells than expected in classical AIH [3,19,22,36,38,48,49]. For this reason, the term “ICI-associated AIH-like immune-mediated hepatitis” is probably more accurate than simply “ICI-induced autoimmune hepatitis” for many patients.

Corticosteroids remain the best-supported treatment in this review, not because high-quality comparative trials exist, but because they are the most consistently used therapy across the available reports. In the present dataset, 102/105 patients treated with corticosteroids alone recovered. This is broadly consistent with current clinical practice and published management recommendations, where corticosteroids are used for clinically significant immune-mediated hepatitis, especially when liver tests worsen, symptoms develop, or grade ≥ 3 toxicity is reached [24,25,28,29,31,44,45,50,51,52,53]. However, the apparent 97.1% response rate should be interpreted with caution. Many studies did not apply uniform response definitions, and patients selected for steroid monotherapy may have differed from those requiring escalation. In addition, biochemical recovery after ICI withdrawal and corticosteroid exposure cannot always be separated from the natural course of the injury. The findings support corticosteroids as first-line therapy, but they do not prove that every patient with aminotransferase elevation requires high-dose steroids.

The issue of steroid dose remains unresolved. In practice, oral prednisone or prednisolone is often used for moderate cases, while intravenous methylprednisolone is used for more severe or non-responding cases. Some guidance recommends escalation if liver tests do not improve within 48–72 h, while other expert recommendations allow a longer interval before second-line treatment, depending on clinical stability [29,50,51,52,53]. Li et al. reported that higher steroid dosing did not clearly improve hepatic outcomes compared with lower-dose regimens, while Zhang et al. suggested that grade 4 hepatitis may require more intensive corticosteroid treatment [7,20,27]. This probably reflects the fact that ICI-hepatitis is not a single biological entity. A patient with asymptomatic aminotransferase elevation and preserved bilirubin and INR is not the same as a patient with jaundice, coagulopathy, or evolving liver failure. The current evidence favours individualised treatment rather than rigid dose escalation based only on aminotransferase thresholds.

MMF was the most consistent second-line agent in steroid-refractory or steroid-resistant cases. In this review, 9/10 patients treated with corticosteroids and MMF recovered, and MMF was also used as part of triple therapy with tacrolimus in severe refractory cases [4,9,13,15,20,21]. This is clinically coherent. MMF suppresses lymphocyte proliferation and is already familiar in autoimmune liver disease and transplant medicine. It also avoids the hepatotoxicity concerns associated with some other immunosuppressants. Current oncology and hepatology guidance generally places MMF as the preferred escalation agent when corticosteroids fail, although the recommended timing varies [29,50,51,52,53]. The data in this review support that position, but again with the caveat that the denominator is small and the cases are not uniform.

Tacrolimus was used less frequently, usually as rescue therapy or as part of multimodal immunosuppression. Zhang et al. and Ziogas et al. reported favourable outcomes when tacrolimus was added to corticosteroids and MMF in refractory disease [20,21]. This makes biological sense, as tacrolimus inhibits calcineurin and T-cell activation. However, tacrolimus requires drug-level monitoring and carries risks of nephrotoxicity, neurotoxicity, hypertension, and infection. Its role should therefore remain selective, particularly for patients with severe disease that does not respond to corticosteroids and MMF, or in centres with experience managing calcineurin inhibitors.

The use of biologics remains more exploratory. Basiliximab was associated with recovery in one severe steroid-refractory case reported by Zarrabi et al. [18]. As an anti-CD25 monoclonal antibody, it targets activated T-cell proliferation and is mechanistically attractive for severe immune-mediated hepatitis. However, one successful case does not establish a treatment pathway. Tocilizumab was used in seven patients in Zhang et al., with recovery in four [20]. This was the lowest response proportion among the extracted treatment groups, although the interpretation is limited by case selection and severity. Infliximab was reported as successful in one patient in the context of steroid-refractory immune-related toxicity [1]. Nevertheless, infliximab remains difficult to endorse broadly in ICI-associated liver injury because anti-TNF therapy itself can be associated with hepatotoxicity and autoimmune-like liver injury. This concern is also supported by broader literature on biological therapies in autoimmune hepatitis and liver injury [60]. Infliximab may be useful for selected non-hepatic irAEs, but its use for hepatitis should remain exceptional and carefully justified.

Liver-directed and supportive therapies were reported in isolated cases. Ursodeoxycholic acid and bezafibrate were useful in the case described by Onishi et al., particularly in a steroid-refractory setting where cholestatic or bile duct features may have contributed to the phenotype [11]. Budesonide was reported in a delayed-onset case and is theoretically attractive because of its high first-pass hepatic metabolism and lower systemic exposure [2]. However, budesonide should not be assumed to be adequate for severe hepatitis, impaired synthetic function, or acute liver failure. DPMAS and plasma exchange were reported in severe cases by Tan et al. and Li et al., with favourable outcomes [7,16]. These approaches may have a role as rescue or bridge therapy in fulminant presentations, particularly when bilirubin, inflammatory mediators, or liver failure physiology dominate the clinical picture. At present, however, they remain case-based interventions rather than standard treatment.

A clinically important finding is that 19 patients recovered without pharmacological treatment [3,4,10,13,19]. This should temper the reflex to treat every liver enzyme elevation aggressively. Some patients with ICI-associated liver injury may improve with ICI interruption, close monitoring, and exclusion of competing diagnoses. The challenge is identifying them safely. CTCAE grading is widely used, but it has limitations in liver disease because it relies heavily on aminotransferases and bilirubin and does not fully capture hepatic function, particularly INR, encephalopathy, or the clinical trajectory [51,53]. A patient with ALT > 5 times the upper limit of normal and normal bilirubin, normal INR, and falling enzymes over 48–72 h is clinically different from a patient with the same ALT and rising bilirubin or coagulopathy. Future work should move beyond enzyme thresholds alone and incorporate trajectory, synthetic function, symptoms, imaging, and competing diagnoses.

The question of ICI discontinuation and rechallenge is central because these patients often have advanced malignancy and may be deriving oncological benefit from treatment. In this review, ICI interruption or discontinuation was reported in 141 patients, and rechallenge was attempted in 55 after recovery. No recurrence was documented among rechallenged patients in the extracted dataset [4,9,10,13,20]. This finding is reassuring but should not be overstated. Rechallenged patients are highly selected. They are usually those who recovered, remained clinically stable, had ongoing oncological indication, and were considered suitable by a multidisciplinary team. The absence of recurrence in this dataset does not mean rechallenge is universally safe. It does suggest that rechallenge can be reasonable in carefully selected patients after biochemical recovery, particularly when the expected cancer benefit is substantial and when monitoring is close.

The safety of immunosuppression is another major theme. The purpose of treatment is to prevent progression of immune-mediated liver injury, but the treatment itself can cause harm. Luo et al. reported deaths among patients treated for steroid-refractory immune-related adverse events, including deaths directly attributed to immunosuppression [9]. Feng et al. described opportunistic infections, including herpes zoster and tuberculosis, during corticosteroid therapy [5]. These reports reinforce a practical point: escalation should be selective. Immunosuppression may be lifesaving in severe hepatitis, but in mild or already improving biochemical injury, high-dose or prolonged treatment can create avoidable harm. This is particularly relevant in patients with advanced cancer, frailty, diabetes, infection risk, viral hepatitis risk, or previous tuberculosis exposure.

The findings also support a more structured diagnostic approach. Before labelling a patient as having ICI-associated AIH-like hepatitis, alternative causes must be actively excluded. Viral hepatitis, hepatitis B reactivation, CMV or EBV infection, biliary obstruction, hepatic metastases, alcohol-related injury, sepsis, vascular disorders, and other hepatotoxic drugs can all mimic or coexist with ICI-hepatitis [24,25,31,35,40,42,45,51]. This matters because management changes when the diagnosis changes. Steroid-refractory hepatitis may reflect an alternative or coexisting diagnosis, including viral reactivation or another drug-induced liver injury. Liver biopsy is not mandatory in every case, but its value increases when the diagnosis is uncertain, hepatitis is severe, corticosteroid response is absent, or autoimmune liver disease or DILI remains plausible [3,19,25,33,36,38,48,49,61,63,64].

From a clinical perspective, management should remain pragmatic and severity-guided. Mild cases may be monitored closely when bilirubin and INR are stable and competing diagnoses have been considered, whereas clinically significant or worsening hepatitis generally warrants ICI interruption and corticosteroids. Failure to improve should prompt diagnostic reassessment, reconsideration of biopsy, and escalation to MMF when appropriate. Tacrolimus, biologics, and extracorporeal liver-support strategies should be reserved for selected severe or refractory cases, ideally with hepatology and oncology input. Rechallenge should be individualised according to the severity of the initial event, completeness of recovery, cancer status, available alternatives, and feasibility of close biochemical monitoring.

The main limitation of this review is the quality and structure of the available evidence. Most included studies were case reports, small case series, retrospective cohorts, or descriptive observational studies, and no randomised controlled trial was identified. Diagnostic terminology, work-up, severity grading, treatment thresholds, and response definitions varied substantially across studies. Autoantibodies, IgG, histology, exclusion of competing causes, and criteria for recovery were also inconsistently reported. Several treatment groups included only one or two patients, making apparent 100% response rates potentially misleading. Rechallenge data were subject to strong selection bias, as only clinically suitable patients were restarted on ICIs. The review was also limited to English-language studies identified primarily through PubMed with manual reference checking, rather than a broader multi-database search, which may have missed relevant reports indexed elsewhere. Finally, because denominators were inconsistent and comparator groups were generally absent, meta-analysis was not feasible. These findings should therefore be interpreted as a descriptive synthesis of the available clinical literature, not as evidence of comparative treatment efficacy.

## 5. Conclusions

ICI-associated autoimmune hepatitis-like liver injury is an important immune-related adverse event in modern oncology. Most cases appear manageable when recognised early, alternative causes of liver injury are excluded, and treatment is guided by clinical severity rather than aminotransferase elevation alone. Corticosteroids remain the main first-line therapy for clinically significant cases, while mycophenolate mofetil is the most consistent second-line option when steroid response is inadequate. Other agents, including tacrolimus, azathioprine, budesonide, ursodeoxycholic acid, bezafibrate, tocilizumab, basiliximab, infliximab, plasma exchange, and DPMAS, have been reported only in selected cases and should remain reserved for refractory or highly individualised scenarios.

The term “autoimmune hepatitis” should be used carefully in this setting. Many cases are better described as AIH-like immune-mediated hepatitis, as they do not consistently reproduce the serological, histological, or clinical phenotype of classical AIH. ICI interruption and rechallenge should also be individualised, since some patients recover without pharmacological treatment and selected patients can restart ICI therapy after resolution without recurrent hepatitis. Rechallenge, however, requires careful patient selection, close biochemical monitoring, and shared decision-making between oncology and hepatology teams.

The current evidence remains limited by small studies, inconsistent definitions, variable diagnostic work-up, and non-standardised treatmentresponse reporting. Future prospective multicentre studies should use uniform criteria for diagnosis, severity, steroid-refractory disease, biochemical recovery, relapse, and rechallenge outcomes. Until stronger data are available, management should remain pragmatic: exclude alternative causes, assess both liver enzymes and hepatic function, treat progressive or clinically significant cases promptly, avoid unnecessary prolonged immunosuppression, and consider ICI rechallenge only when the expected oncological benefit justifies the risk.

## Figures and Tables

**Table 1:** Summary of included studies, patient counts, treatment characteristics, and ICI rechallenge outcomes.

Study	Total Patients	Grade ≥ 3 Cases	Resolved Without Steroids	ICI Stopped	ICI Restarted
De Martin, 2018 [4]	16	16	6	16	16
Kocheise, 2023 [8]	3	No	No	No	No
Zhang, 2024 [20]	51	51	No	51	12
Jilkova, 2021 [22]	16	No	No	No	No
Riveiro-Barciela, 2023 [13]	23	23	4	23	23
Luo, 2021 [9]	6	NA	0	4	1
Sanz-Segura, 2021 [14]	4	NA	NA	NA	NA
Yamamoto, 2021 [17]	21	NA	NA	NA	NA
Coukos, 2022 [3]	26	26	3	26	No
Zen, 2018 [19]	6	1	1	1	No
Purde, 2022 [10]	11	5	5	9	3
Zarrabi, 2023 [18]	1	1	No	Yes	No
Parakh, 2018 [12]	1	1	No	Yes	No
Li, 2022 [7]	2	2	No	Yes	No
Araujo, 2021 [1]	1	1	No	Yes	No
Ziogas, 2020 [21]	1	1	No	Yes	No
Onishi, 2020 [11]	1	1	No	Yes	No
Feng, 2022 [5]	1	1	No	Yes	No
Honma, 2021 [6]	1	1	No	Yes	No
Tan, 2021 [16]	1	1	No	Yes	No
Shah, 2019 [15]	1	1	No	Yes	No
Chopra, 2023 [2]	1	1	No	Yes	No
—	195	134	19	141	55

**Table 2:** Treatment modalities and recovery rates among patients with ICI-associated autoimmune hepatitis-like liver injury or immune-mediated hepatitis.

Treatment Modality	No. Recovered/No. Treated	Recovery Rate (%)
Corticosteroids alone [3,4,6,10,12,13,14,17,19,20]	102/105	97.1
Corticosteroids + mycophenolate mofetil [4,9,13,15,20]	9/10	90
Corticosteroids + mycophenolate mofetil + tacrolimus [20,21]	3/3	100
Infliximab [1]	1/1	100
Ursodeoxycholic acid + bezafibrate [11]	1/1	100
DPMAS ± plasma exchange [7,16]	2/2	100
Ursodeoxycholic acid [6]	1/1	100
Corticosteroids + azathioprine [8]	1/1	100
Corticosteroids + tocilizumab [20]	4/7	57
Basiliximab [18]	1/1	100
Corticosteroids + unspecified immunosuppressive agents [3]	7/7	100
Oral budesonide [2]	1/1	100

## Data Availability

No new data were created or analyzed in this study.

## Supplementary Materials

The following supporting information can be downloaded at: https://www.mdpi.com/article/10.3390/diagnostics16121821/s1, PRISMA 2020 Checklist. Table S1. Joanna Briggs Institute’s (JBI) critical appraisal checklist for Case reports. Table S2. Robvis risk of bias assessment-Traffic light plot for Case reports. Table S3. Robvis risk of bias assessment-Summary plot for Case reports. Table S4. Joanna Briggs Institute’s (JBI) critical appraisal checklist for Prevalence studies. Table S5. Robvis risk of bias assessment-Traffic light plot for Prevalence studies. Table S6. Robvis risk of bias assessment-Summary plot for Prevalence studies. Table S7. Characteristics of studies selected for review.
