# Supplementary material for: Autoimmune Hepatitis Induced by Immune Checkpoint Inhibitors in Adults: A Systematic Review
Source: Diagnostics (Basel). 2026 Jun 12;16(12):1821. doi: 10.3390/diagnostics16121821 (PMC13298183; doi:10.3390/diagnostics16121821)
Supplement: Supplementary file 1 [file diagnostics-16-01821-s001.zip › diagnostics-4318728-supplementary.pdf]

Supplementary Materials

List of checklists/tables

Table S1. Joanna Briggs Institute’s (JBI) critical appraisal checklist for Case reports

Table S2. Robvis risk of bias assessment - Traffic light plot for Case reports

Table S3. Robvis risk of bias assessment - Summary plot for Case reports

Table S4. Joanna Briggs Institute’s (JBI) critical appraisal checklist for Prevalence studies

Table S5. Robvis risk of bias assessment - Traffic light plot for Prevalence studies

Table S6. Robvis risk of bias assessment - Summary plot for Prevalence studies

Table S7. Characteristics of studies selected for review

Study Title	Demographics?	History & Timeline	Diagnostic Tests or Assessment	Intervention or Treatment	Post-intervention Clinical	Adverse Events	Key Takeaway?	Overall Risk of Bias
Zarrabi, 2023	Low	Low	Low	Low	Low	High	Low	Low
Parakh S, 2018	Low	Low	Low	High	Low	Low	Low	Moderate
Li X, 2022	Low	Low	Low	Low	Low	High	Low	Low
Ziogas D C, 2020	Low	Low	Low	Low	Low	Low	Low	Low
Sachiyo Onishi, 2020	Low	Low	Low	Low	Low	High	Low	Low
Yi Feng, 2022	Low	Low	Low	Low	Low	High	Low	Low
Honma, 2021	Low	Low	Low	Low	Low	Low	Low	Low
You-Wen Tan, 2021	Low	Low	Low	Low	Low	High	Low	Low
N Shah, 2019	Low	Low	Low	Low	Low	High	Low	Low
Chopra, 2023	Low	Low	Low	Low	Low	High	Low	Low

Table S1 Joanna Briggs Institute's (JBI) critical appraisal checklist for Case reports

1. Were patient's demographic characteristics clearly described?
2. Was the patient's history clearly described and presented as a timeline?
3. Was the current clinical condition of the patient on presentation clearly described?
4. Were diagnostic tests or assessment methods and the results clearly described?
5. Was the intervention(s) or treatment procedure(s) clearly described?
6. Was the post-intervention clinical condition clearly described?
7. Were adverse events (harms) or unanticipated events identified and described?
8. Does the case report provide takeaway lessons?

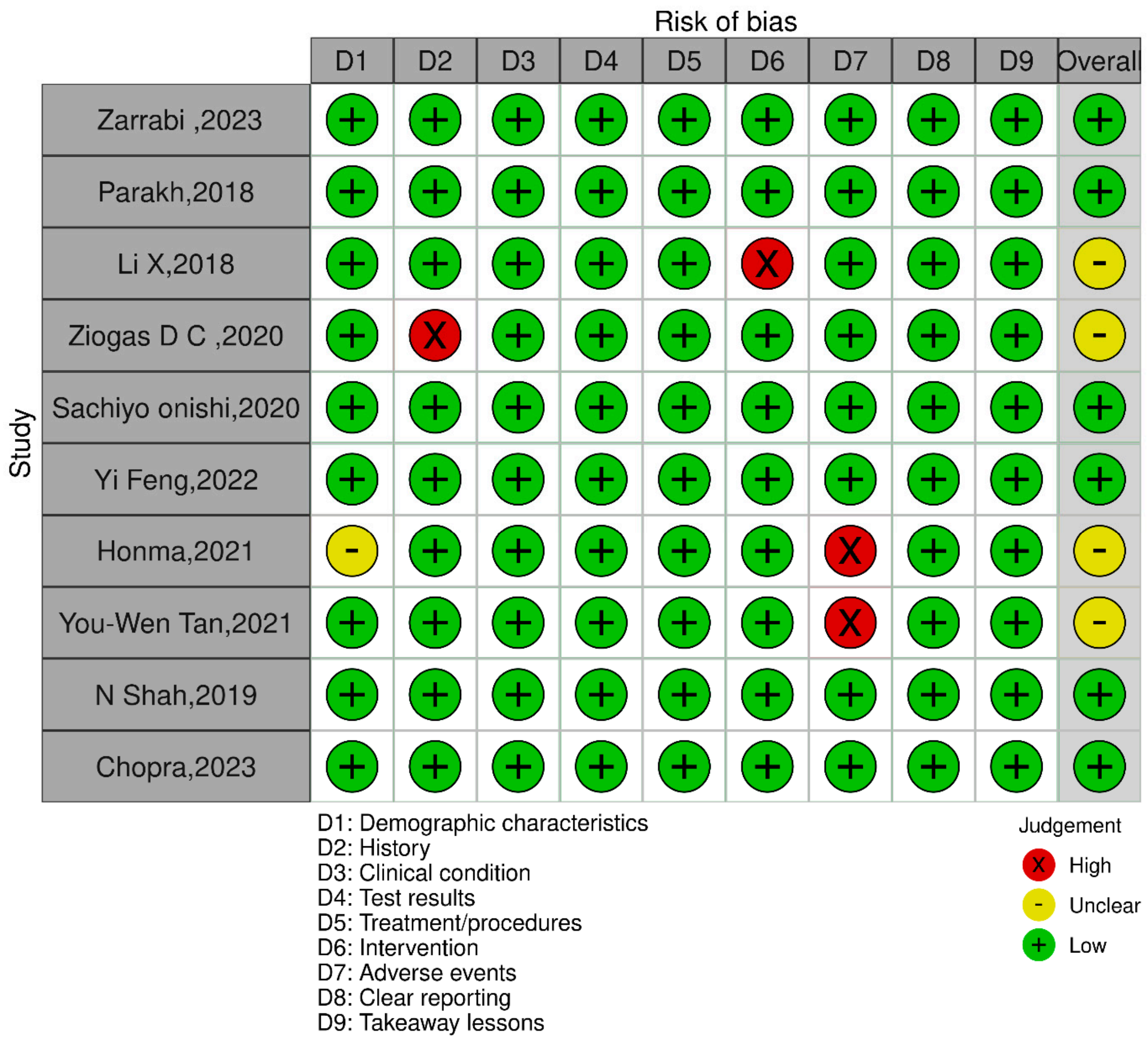

Table S2. Robvis risk of bias assessment - Traffic light plot for Case reports

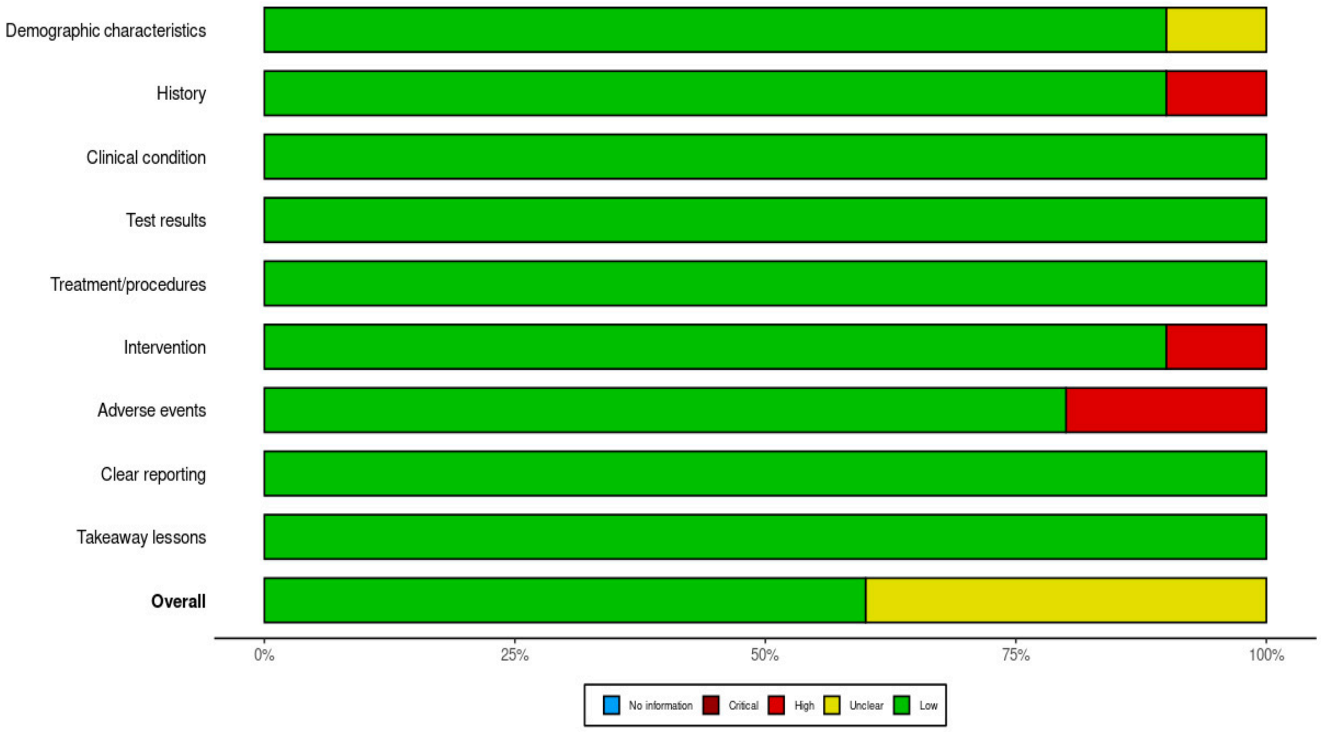

Table S3 Robvis risk of bias assessment - Summary plot for Case reports

Study	Sample frame	Study participants	sample size	study subjects and setting	Data analysis	Methods for identification	Condition measured appropriately	Statistical analysis	Response rate	Overall
De Martin et al., 2018	Low	Low	Low	Low	Low	Low	Low	Low	Low	Low
Kocheise et al., 2024	Unclear	Low	Low	Low	Low	Low	Unclear	Low	Low	Low
Zhang et al., 2024	Low	Low	Low	Unclear	Low	Low	Low	Low	Low	Some concerns
Zuzana Macek Jilkova et al., 2021	Low	Low	Low	Unclear	Low	Unclear	High	Unclear	Low	Some concerns
Mar Riveiro - Barciela et al., 2023	High	High	High	Low	Low	Unclear	Unclear	High	Low	Some concerns
Patricia Sanz-Segura et al., 2021	Low	High	High	Unclear	Low	Low	Unclear	High	Low	High
Atsushi Yamamoto et al., 2021	Low	Unclear	Unclear	Unclear	High	Low	No information	High	Low	High
Aude Coukos et al., 2022	Low	Unclear	Unclear	Low	Low	Low	Low	Unclear	Low	Some concerns
Yoh Zen et al., 2018	Low	Low	Unclear	High	Unclear	Unclear	Low	High	Low	Some concerns
Purde MT et al., 2022	Low	Low	Low	Low	Low	Low	Low	Low	Low	Low
Lou et	Low	Low	Low	Uncle	Uncl	Low	Low	Uncle	Low	Some

al., 2021				ar	ear			ar		conce rns
Aroujo et al., 2021	Low	Low	Low	Low	Low	Low	Low	Low	Low	Low

Table S4 Joanna Briggs Institute's (JBI) critical appraisal checklist for Prevalence studies

JBI Checklist for Prevalence studies:

- Was the sample frame appropriate to address the target population?
- Were study participants sampled appropriately?
- Was the sample size adequate?
- Were the study subjects and the setting described in detail?
- Was the data analysis conducted with sufficient coverage of the identified sample?
- Were valid methods used for the identification of the condition?
- Was the condition measured in a standard, reliable way for all participants?
- Was there appropriate statistical analysis?
- Was the response rate adequate, and if not, was the low response rate managed appropriately?

Risk of bias

Study	D1	D2	D3	D4	D5	D6	D7	D8	D9	Overal
De Martin et al;2018	+	+	+	+	+	+	+	+	+	+
Kocheise et al;2024	-	+	+	+	-	+	-	+	+	+
Zhang et al;2024	+	+	+	-	+	+	+	+	+	-
Zuzana Macek Jilkova et al, 2021	+	+	+	+	+	+	X	-	+	+
Mar Riveiro-Barciela et al, 2023	X	X	X	-	-	+	-	+	+	+
Patricia Sanz-Segura et al, 2021	+	X	X	-	+	+	-	+	-	X
Atsushi Yamamoto et al, 2021	+	-	-	-	X	+	?	X	+	X
Aude Coukos et al, 2022	+	-	-	+	+	+	+	-	+	+
Yoh Zen et al, 2018	+	+	-	X	-	-	+	X	+	+
Purde MT et al, 2022	+	+	+	+	+	+	+	+	+	+
Lou et al2021	+	+	+	-	-	?	+	-	-	-
Aroujo et al,2021	+	+	+	+	+	?	+	+	-	+

D1: Sample frame
D2: sampling method
D3: Sampling size
D4: Setting description
D5: Analysis coverage
D6: Valid identification method
D7: measurement consistency
D8: statistical anlysis
D9: esponse rate

Judgement
X High
- Unclear
+ Low
? No information

Table S5 Robvis risk of bias assessment - Traffic light plot for Prevalence studies

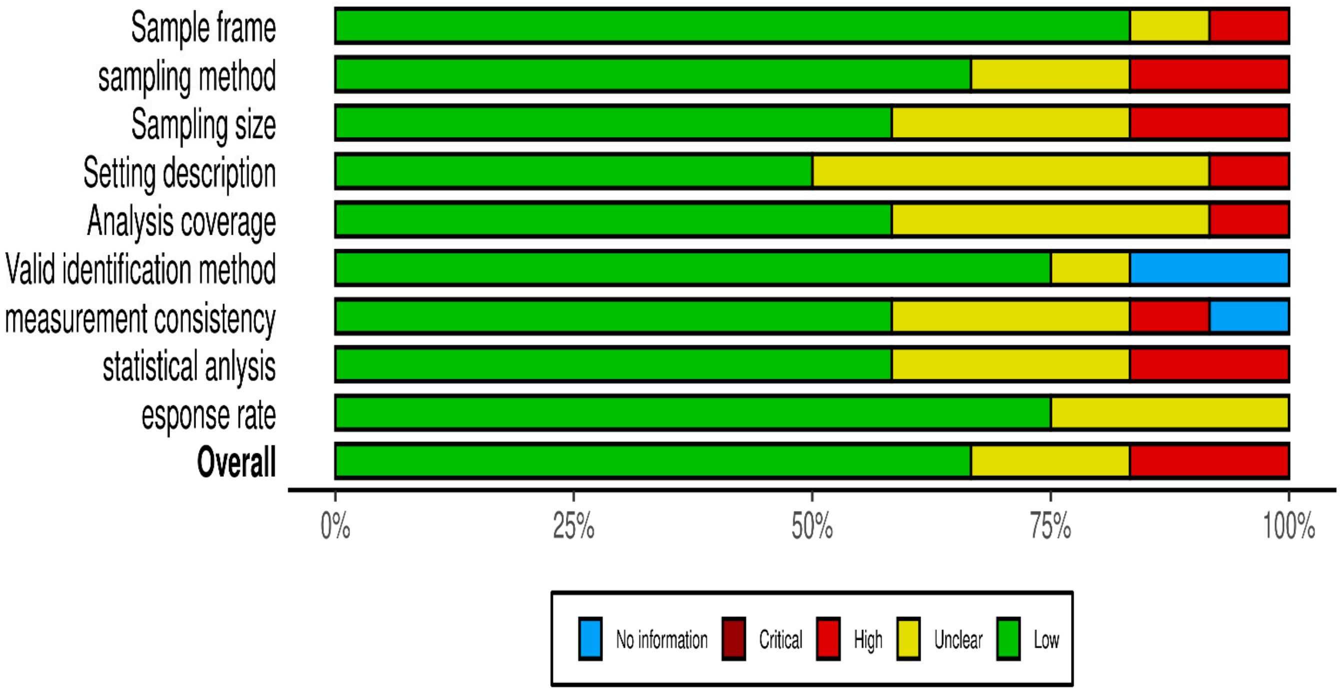

Table S6 Robvis risk of bias assessment - Summary plot for Prevalence studies

Table S7 Characteristics of included studies

Name of the study	Last name of author and Year of publication	Country of Study	Study design and setting	Number of patients with AIH enrolled	Patient population summary	Intervention used	Treatment used for AIH due to ICI	Rate of response to treatment	Definition of outcomes measured	Number of patients who achieved the outcome in intervention group	Number of patients who achieved the outcome in the comparison group	ICI reintroduced / Liver dysfunction recurrence
**Characterization of liver injury induced by cancer immunotherapy using immune checkpoint inhibitors**	De Martin, 2018	France	Retrospective observational study; The study was conducted at a tertiary academic medical centre in France, primarily focusing on patients having ICI for cancer treatment	16	The patients included were those who developed liver injury after treatment with ICI. These patients were treated for various cancers including melanoma, NSCLC, renal cell, etc. The study examined the	use of immune checkpoint inhibitors (ICIs), specifically anti-PD-1/PD-L1 (such as nivolumab, pembrolizumab) and anti-CTLA-4 (such as ipilimumab)	7 patients--> oral corticosteroids 0.5-1 mg/kg/day, 2 patients -->0.2 mg/kg/day maintenance,1 patient -->required pulses and 2.5 mg/kg/day of corticosteroids and addition of second immunosuppressant	9/10 full resolution with steroids alone	Primary outcome: The liver injury caused due to ICI was characterised according to the liver enzymes (AST/ALT), severity of liver injury (mild to moderate). Resolution of the injury after treatment with steroids, or other interventions. It also examined the outcomes related to liver	resolution of liver injury was achieved in almost all the patients, though the exact numbers varied.50% of patients with severe liver injury had partial resolution after treatment cessation and corticosteroid therapy.	There was no formal comparison group	ICI reintroduced in 3 patients with no occurrence of liver dysfunction

					characteristics of liver injury associated with these therapies, and the patient population had undergone treatment with anti-PD-1/PD-L1 and/or anti-CTLA-4 monoclonal antibodies				toxicity			
**PD-1/PD-L1 immune checkpoint therapy demonstrates favourable safety profile in patients with autoimmune and cholestatic liver disease**	Kocheise,2024	Multiple European countries including Germany, France, Spain, Netherlands,	Retrospective multicentre cohort study	3	The study included patients with autoimmune liver disease who were treated with ICI for various cancers with the following breakdown:PBC-12 patients, PSC-5 patients, AIH-4 patients, AIH-PSC variant -1 patient. Malignancies included hepatobiliary and non-	Atezolizumab (7), Durvalumab(5), Pembrolizumab (4), Nivolumab (4), Spartalizumab(1), Combination therapy (Nivolumab + Ipilimumab) : 1	1 patient without previous AIH was given Ursodeoxycholic acid. 1patient with previous AIH on MMF was given oral prednisolone 15 mg/day, and the third patient with AIH on Azathioprine and Predisolone, the dose of Prednisone was increased to 10 mg/day and then reduced to 7.5 mg /day after 3 weeks after liver enzymes normalised.	All 3 patients had normalisation of liver enzymes within 3 weeks	Primary outcome: Safety of PD-1/PD-L1 inhibitors in patients with AILD, assessed by the occurrence and severity of immune-related adverse events, particularly liver-related irAEs. Secondary outcome: Changes in liver function tests during the first year of ICI treatment	Out of 3 patients with Liver irH due to ICI, 2 patients had previous AIH and were on immunosuppressive drugs before treatment started. 1 patient did not have AIH. All 3 patients had resolution of liver dysfunction after 3 weeks of starting the treatment drugs and stopping ICI	There was no formal comparison group	ICI reintroduced in all 3 patients and successfully completed treatment without liver function derangement.

					hepatic tumours							
**Management and treatment of severe immune related hepatotoxicity based on clinical and pathological characteristics**	Zhang, 2024	China	Multicentre retrospective study; involved patients from Peking Union Medical College Hospital and Cancer Hospital, Chinese Academy of Medical Sciences. Patients who developed severe irH and those without irH after immunotherapy were enrolled	51	5,326 patients received immune checkpoint inhibitors (ICPis), among which 186 developed immune-related hepatotoxicity (irH), and 51 developed severe irH(> =grade 3). The severe irH group included 51 patients with a median age of 65 years (IQR 57–68), predominantly male (72.5%). The most common primary tumours were lung cancer (39.2%) and	All patients with severe irH discontinued ICPi therapy and were treated with glucocorticoids	All patients were treated with Glucocorticoids. 2 needed addition of MMF and Tacrolimus and 7 patients needed addition of Tocilizumab	out of 51 patients, 42 patients resolved with steroids. Out of 9 patients 6 patients resolved within 3 months; 3 died	Time to resolution (TTR), defined as the time between the administration of GCS and the normalisation of liver function	All patients with grade 3 irH were steroid-sensitive and completely cured with steroids; 21 patients with grade 4 responded to steroids alone. 2 patients who were steroid-resistant were treated with MMF and Tacrolimus; 7 patients were given Ivi and Tocilizumab. Out of the 9, 6 patients achieved cure within 3 months and 3 patients died.	Not specified in the provided information.	12 patients were retreated with Ici with no recurrence of liver derangement

					hepatobiliary carcinoma (15.7%)							
**Increased intrahepatic expression of immune checkpoint molecules in autoimmune liver disease**	Zuzana Macek Jilkova, 2021	France	Cross-sectional, observational study conducted at the Department of Gastroenterology and Hepatology, CHU Grenoble-Alpes, France.	16	The AIH cohort comprised individuals diagnosed with autoimmune hepatitis, with 11 untreated and 5 treated patients. The control group included patients with suspected non-alcoholic fatty liver disease, who were later excluded upon confirmation of the diagnosis.	No therapeutic intervention			The primary outcome was the expression of immune checkpoint molecules. Secondary outcomes included the correlation between ICM expression and liver enzyme levels (ALT, AST) as indicators of disease severity.	Observational study without an intervention; there were no specific outcomes in the intervention group	No outcomes to achieve	
**Retreatment With Immune Checkpoint Inhibitors After a Severe Immune-**	Mar Riveiro-Barciela, 2023	Spain	Prospective, multicenter, non-interventional study conducted across three academic	23	The cohort included patients with various cancers, with the most	Retreatment with ICIs, primarily the same ICI previously administered, after a	19 patients received corticoids--> 12 received oral prednisone and 7 received methylprednisolone. Out of	17 patients responded to steroids and 2 patients responded to MMF+steroids.	The primary outcome was the recurrence of immune-related hepatitis after ICI retreatment.	15 out of 23 patients did not experience recurrence of immune-related hepatitis after retreatment	Not applicable	23 patients were retreated with ICI. 8

**Related Hepatitis: Results From a Prospective Multicenter Study.**			hospitals in Spain		frequent being lung and urinary tract cancers. All patients had previously experienced severe immune-related hepatitis due to ICIs and had their immunotherapy discontinued.	median of 10 weeks from the resolution of the hepatitis	these 2 patients needed MMF as second-line immunosuppression.		Secondary outcomes included the need for permanent discontinuation of ICIs and the reasons for discontinuation			patients developed irH. In these patients 6 patients -ICI was stopped permanently and 2 patients it was restarted after a short time. 1 patient developed acute liver failure which responded well to plasma exchange.
**Beyond steroids:Immunosuppressants in steroid refracrtory or resistant immune related**	Luo J, 2021	United States	Retrospective cohort study conducted at Memorial Sloan Kettering Cancer Center (MSKCC), New York, USA.	6	The cohort included patients with advanced lung cancers treated	The study evaluated the use of additional immunosuppressants in patients who were refractory	6 patients with Hepatitis received MMF with steroids	5 / 6 patients showed (83%) showed improvement in liver function in 3 months.	The primary outcome was the clinical improvement of the irAE at 90 days following the initiation of the additional	5 out of 6 patients with Hepatitis showed resolution of their hepatitis	Not applicable	1 patient with hepatitis due to ICI was rechal

**adverse events**					with immune checkpoint inhibitors between 2011 and 2020. The median age was 64 years, with 57% female and 43% male. Most patients (84%) were former or current smokers. The majority had non-small cell lung cancer (NSCLC), with 71% having adenocarcinoma histology	or resistant to steroids for managing severe irAEs. Most commonly used agents TNF-alpha and Mycophenolate Mofetil. Patients with Pneumonitis and Colitis received Infliximab, and those with hepatitis received MMF.			immunosuppressant. Improvement was defined as a reduction in symptoms and stabilisation or recovery of organ function.			lenged with ICI and there was no recurrence after 39 months.
**Gastrointestinal and liver immune-related adverse effects induced by immune checkpoint inhibitors: A descriptive observational study.**	Patricia Sanz-Segura, 2021	Spain	Retrospective, single-centre, observational study conducted at the Miguel Servet University Hospital in Zaragoza, Spain	4	The cohort included patients with various types of advanced cancer, including non-small cell lung cancer (NSCLC), melanoma, and renal	Administration of ICI- Nivolumab, Pembrolizumab, Atezolizumab, durvalumab, Ipilimumab, and a combination of anti-CTLA4 and anti-PD1	Steroids needed in 2 patients	1 patient recovered	The primary outcomes were the incidence and severity of gastrointestinal (GI) and liver immune-related adverse events (IrAEs), classified according to the Common Terminology	1 patient recovered after steroids and 1 patient had to discontinue treatment	Not applicable	1 patient continued treatment after rechallenge and the other had to discontinue

					cell carcinoma				Criteria for Adverse Events (CTCAE) version 4.0. GI-IrAEs were further categorised into grades 1 to 4 based on severity.4 patients developed Li-irAEs			treatment.
**Clinical fetures of immune mediated hepatotoxicity induced by ICI in patients with cancers**	Atsushi Yamamoto, 2021	Japan	Retrospective, single-centre, observational study conducted at Kobe University Graduate School of Medicine, Japan	21	The cohort included 202 males and 48 females, with a median age of 71 years (range 30–87). The study focused on patients who developed elevated transaminase levels (>3× upper limit of normal) during ICI therapy	Immune checkpoint inhibitors, including ipilimumab, nivolumab, pembrolizumab, and atezolizumab	Steroids given to 13 patients	13/13 patients recovered	The primary outcome was the development of immune-mediated hepatotoxicity (IMH), defined as elevated transaminase levels (>3× upper limit of normal) during ICI therapy	21 Patients	Not applicable	None of the patients were retreated with ICI
**Systematic comparison with autoimmune liver disease identifies specific histological**	Aude Coukos, 2022	Switzerland	Retrospective, multicenter, observational study conducted across multiple Swiss hospitals	26	The cohort comprised patients who developed liver-related immune	Immune checkpoint inhibitors, including nivolumab, pembrolizumab, and ipilimumab	Steroids alone -->7, Steroids +Other immunosuppressive agents -->7	7 out of 14 patients responded to steroids alone. Remaining 7 patients responded to other immunosuppress	The primary outcome was the identification of histological features specific to ICI-induced liver irAEs	Not applicable as the study focused on histological comparisons	Not applicable	

**features of immune checkpoint inhibitor-related adverse events**					checkpoint inhibitor (ICI)-induced adverse events (irAEs) and were compared with patients diagnosed with autoimmune liver diseases (AILDs)			ive agents along with steroids	compared to AILDs			
**Hepatotoxicity of immune checkpoint inhibitors: a histology study of seven cases in comparison with autoimmune hepatitis and idiosyncratic drug-induced liver injury.**	Yoh Zen, 2018	Japan	Retrospective histological study conducted at Kobe University Graduate School of Medicine	6	Patients who developed liver dysfunction after treatment with immune checkpoint inhibitors (nivolumab or ipilimumab)	Immune checkpoint inhibitors (nivolumab and ipilimumab)	4 patients received Methyprednisolone at 100 mg /day for 3 days, 1 patient received Prednisolone 30 mg/day for 26 days and 1 patient received 50 mg /day for 13 days.	5 patients showed good response with normalisation of liver enzymes	Histological features of liver injury	All 7 patients had histological features of liver injury.1 patient continued to have mild liver function abnormality in spite of steroids.	: Not applicable	
**Presence of autoantibodies in serum does not impact the occurrence of immune checkpoint inhibitor-induced hepatitis in a prospective**	Purde MT, 2022	Switzerland	Prospective, multicenter, observational cohort study conducted at Kantonsspital St. Gallen, Switzerland	11	Patients receiving immune checkpoint inhibitors (ICIs) between July 2016 and May 2019	Immune checkpoint inhibitors (e.g., nivolumab, pembrolizumab, ipilimumab	6 patients received Steroids	All 6 patients showed normalisation of LFT's within few weeks	Development of ICI-induced hepatitis, defined by elevated liver function tests (LFTs)	11 patients developed ICI-induced hepatitis	None	ICI restarted in 3 patients. No further deterioration in liver enzymes.

**cohort of cancer patients**												
**Successful treatment of severe immune checkpoint inhibitor associated autoimmune hepatitis with basiliximab: a case report.**	Maiah Zarrabi, 2023	United States	Case report from the University of California, Los Angeles (UCLA) Mattel Children's Hospital	1	The patient developed severe autoimmune hepatitis after receiving nivolumab, an anti-PD-1 therapy, for refractory osteosarcoma	Basiliximab (anti-CD25 monoclonal antibody)	Basiliximab 20 mg/dose - 2 doses in first week followed by weekly doses of total 6 doses while continuing on steroids, MMF and Tacrolimus. Then was weaned off steroids and MMF and Tacrolimus was discontinued.	1 patient achieved sustained resolution of ICI-induced hepatitis (100%)	Resolution of liver function test abnormalities (ALT, AST, GGT) and clinical symptoms	1 patient achieved sustained resolution of hepatitis without significant side effects	Not applicable	ICI not restarted
**Delayed Autoimmune Toxicity Occurring Several Months After Cessation of Anti-PD-1 Therapy.**	Parakh S, 2018	Australia	Case report from the Medical Oncology Unit at Austin Health and the Olivia Newton-John Cancer Research Institute in Heidelberg, Victoria, Australia	1	The patient developed delayed autoimmune hepatitis 8 months after discontinuing nivolumab therapy	High-dose methylprednisolone (2 mg/kg) followed by oral prednisolone (1 mg/kg)	High-dose Methyprednisolone 2 mg/kg followed by oral Prednisolone 1 mg/kg	1 patient achieved resolution of LFT and clinical symptoms (100%)	Resolution of liver function abnormalities (ALT, AST, GGT) and clinical symptoms	1 patient achieved complete resolution of hepatitis without significant side effects	Not applicable	ICI not restarted
**Application of artificial liver in immune-related liver injury induced by immune checkpoint inhibitor: Case**	Li X, 2022	China	Case reports and literature review conducted at Shanxi Bethune Hospital, Shanxi Academy of Medical Sciences,	2	Patients with metastatic gastrointestinal malignancies who developed severe immune-mediated	Non-biological artificial liver therapy, including plasma exchange and double plasma molecular	Non-biological artificial liver therapy including plasma exchange and double plasma molecular adsorption system combined with	2 patients achieved restoration of liver function and continued with anti-cancer treatment (100%)	Resolution of liver function abnormalities (ALT, AST, bilirubin levels) and clinical symptoms	2 patients achieved restoration of liver function and continued anti-tumour treatment	Not applicable	

**reports and review of the literature.**			Taiyuan, China		hepatotoxicity after receiving immune checkpoint inhibitors (ICIs)	adsorption system (DPMAS), combined with immunosuppressive treatment (glucocorticoids and mycophenolate mofetil)	Glucocorticoids and Mycophenolate Mofetil				
**Real World Outcomes and Hepatotoxicity of Infliximab in the Treatment of Steroid-Refractory Immune-Related Adverse Event**	Daniel V. Araujo, 2021	Canada	Retrospective cohort study conducted at Princess Margaret Cancer Centre, Toronto, Canada	1	Patients with cancer who developed steroid-refractory immune-related adverse events (irAEs) after receiving immune checkpoint inhibitors (ICIs)	Immune checkpoint inhibitors	Infliximab	Complete resolution in 1 patient	Resolution of irAEs, liver function tests (ALT, AST, total bilirubin), recurrence of irAEs, overall survival	1 patient with hepatitis and 43 /51 patients of total patients with irAEs resolved	Not applicable
**When steroids are not enough in immune-related hepatitis: current clinical challenges discussed on the basis of a case report**	Ziogas D.C., 2020	Greece	Case report and literature review conducted at Laiko General Hospital, Athens, Greece	1	A 70-year-old male with metastatic melanoma who developed severe steroid-resistant immune-related hepatitis after treatment with ipilimumab	Ipilimumab	Tacrolimus, MMF, Steroids	1 patient achieved complete resolution of liver derangement (100%)	Resolution of liver function abnormalities (ALT, AST, bilirubin levels) and clinical symptoms	1 patient achieved complete resolution of hepatitis	Not applicable

**Ursodeoxycholic acid and bezafibrate were useful for steroid-refractory, immune related hepatitis: a case report**	Sachiyo Onishi, 2020	Japan	Case report conducted at Aichi Cancer Center Hospital, Nagoya, Japan	1	A 68-year-old male with metastatic melanoma who developed steroid-refractory immune-related hepatitis after receiving nivolumab	Nivolumab	Ursodeoxycholic acid and Bezafibrate.	Improvement in liver function	Improvement in liver function tests (ALT, AST, bilirubin levels) and clinical symptoms	1 patient achieved complete resolution of hepatitis	Not applicable
**Nivolumab Combined With Ipilimumab Treatment Induced Hypophysitis and Immune-Mediated Liver Injury in Advanced Esophageal Squamous Cell Carcinoma: A Case Report**	Yi Feng, 2022	China	Case report conducted at Zhongshan Hospital, Fudan University, Shanghai, China	1	A 55-year-old male with advanced oesophageal squamous cell carcinoma (stage IV)	Combination of nivolumab (3 mg/kg) and ipilimumab (1 mg/kg)	Corticosteroids	Patient developed Herpes Zoster and TB and hence steroids were suspended. The patient continued to have grade 2 Liver failure	Development of hypophysitis and immune-mediated liver injury	1 patient developed both hypophysitis and immune-mediated liver injury	Not applicable
**Rapid Progression of Liver Fibrosis Induced by Acute Liver Injury Due to Immune-related Adverse Events of Atezolizumab**	Honma, 2021	Japan	This is a case report conducted at a Japanese medical institution, as indicated by the authors' affiliations.	1	The patient had no prior history of acute or chronic liver diseases, did not smoke, and had no history of habitual alcohol	The patient received systemic chemotherapy comprising atezolizumab (1, 200 mg/body), bevacizumab (15 mg/kg body weight), carboplatin, and	Corticosteroids	Improvement in Liver function	The primary outcomes measured were the progression of liver fibrosis and the resolution of liver injury, assessed through sequential liver biopsies and monitoring of	Since this is a case report, the outcomes pertain to the single patient involved. The intervention led to improvement in liver injury markers and histopathological findings, but also revealed rapid progression of liver fibrosis.	Not applicable

					consumption. She started a chemotherapy regimen that included atezolizumab, bevacizumab, carboplatin, and paclitaxel	paclitaxel every four weeks as first-line treatment for three months. Upon developing liver injury, corticosteroid therapy with oral prednisolone (45 mg/day) was initiated, along with an increased dose of ursodeoxycholic acid (UDCA) (600 mg/day).			serum liver enzymes and fibrosis markers		
**Efficacy of artificial liver support system in severe immune associated hepatitis caused by camrelizumab: A case report and review of the literature**	You-Wen Tan, 2021	China	This is a case report conducted at The Third Hospital of Zhenjiang, affiliated with Jiangsu University, located in Zhenjiang, Jiangsu Province, China	1	The patient is a 75-year-old man who had no prior history of hepatitis A-E, alcoholism, or other liver diseases. He had been treated with capecitabine and camrelizumab for lymph	The patient received dual plasma molecular adsorption system (DPMAS) combined with plasma exchange (PE) after developing acute liver failure	Sequential dual-molecule plasma adsorption system (DPMAS) treatment with plasma exchange (PE)	Full resolution of liver function in 1 patient	The primary outcomes measured were the improvement in liver function, assessed through laboratory tests such as total bilirubin levels, and clinical symptoms like appetite and urine colour	The single patient involved showed significant improvement in liver function and clinical symptoms after the intervention	Not applicable

					node metastasis of oesophageal cancer						
**Unchecked immunity: a unique case of sequential immune-related adverse events with Pembrolizumab**	N Shah, 2019	Australia	This is a case report conducted at St George Hospital in Kogarah, New South Wales, Australia	1	The study involved a single patient, a 70-year-old male diagnosed with metastatic melanoma. He had a medical history including type 2 diabetes mellitus, hypertension, dyslipidaemia, and atopic dermatitis. His melanoma was classified as stage 4 M1c disease, with metastases in the left inguinal region, liver, and brain. The patient initially received BRAF/ME	The patient was treated with pembrolizumab (2 mg/kg every 3 weeks) as a single-agent anti-PD-1 therapy. Upon developing immune-related adverse events (irAEs), immunosuppressive treatments were administered, including high-dose oral glucocorticoids tapered over a 2-month period, mycophenolate mofetil, high-dose oral Prednisolone, Intravenous Immunoglobulin	Mycophenolate and high-dose oral corticosteroids	Biochemical resolution in 1 patient (100%)	The primary outcomes measured were the resolution of immune-related adverse events, assessed through clinical improvement and laboratory tests such as liver function tests and kidney function markers. Secondary outcomes included the patient's cognitive function and quality of life, evaluated through clinical assessments and cognitive screening	The single patient involved showed significant improvement in liver and kidney function, as well as cognitive function, following the intervention. Biochemical resolution of autoimmune hepatitis was achieved once off glucocorticoids, and cognitive screening demonstrated frontal and executive impairment with corresponding behavioural symptoms thought to be due to prior frontal lobe surgery, exacerbated by high-dose steroid treatment	Not applicable

					K inhibitors, followed by single-agent pembrolizumab therapy	therapy for 5 days.						
**Recognition of Delayed-Onset Immune Checkpoint Inhibitor-Mediated Hepatotoxicity Treated With Budesonide: A Case Study Factoring in Pharmacokinetics of Immunotherapies**	Chopra, Oct 2023	US	This is a case study of patient treated at University of Texas MD Anderson Cancer Center, Houston, TX	1	The study involves a single patient who was treated with RT and Pembrolizumab followed by Ipilizumab and Nivolumab for stage 4 Melanoma. Patient presented with lower abdominal discomfort and nausea with raised transaminases 11 months after stopping the treatment.	Patient was treated with oral Budesonide starting with 9 mg/day and tapering slowly to 6 mg/day and then 3 mg/day for a total of 42 days, and adjunct Ursodeoxycholic acid.	Oral Budesonide tapering dose of 9 mg/day then 6 and then 3 mg/day for total of 42 days.	Adequate biochemical remission	Biochemical resolution and achievement of baseline results	1 patient achieved adequate response	NA	NO
